# 104. Improving Efficiency of Antimicrobial Stewardship Reviews Using Artificial Intelligence Modelling

**DOI:** 10.1093/ofid/ofab466.306

**Published:** 2021-12-04

**Authors:** Si Lin Sarah Tang, Winnie Lee, Yiling Chong, Akshay Saigal, Peijun Yvonne Zhou, Kai Chee Hung, Lun Yi Tan, Shimin Jasmine Chung, Lay Hoon Andrea Kwa

**Affiliations:** 1 Singapore General Hospital, Singapore, Not Applicable, Singapore; 2 DXC Technology, Singapore, Not Applicable, Singapore

## Abstract

**Background:**

Antimicrobial stewardship programs (ASP) in hospitals improve antibiotic prescribing, slow antimicrobial resistance, reduce hospitalisation duration, mortality and readmission rates, and save costs. However, the strategy of prospective audit and feedback is laborious. In Singapore General Hospital (SGH), 10 reviews are required to identify 2 inappropriate cases. Limited manpower constraints ASP audits to only about 30% of antibiotics prescribed. This proof-of-concept study explored the feasibility of developing a predictive model to prioritise inappropriate antibiotic prescriptions for ASP review.

**Methods:**

ASP-audited adult pneumonia patients from January 2016 to December 2018 in SGH were included. Patient data e.g., demographics, allergies, past medical history, and relevant laboratory investigations at each antibiotic use episode were extracted from electronic medical records and re-assembled through linking for analysis. Ground truth for model training was based on ASP-defined appropriateness for each encounter. The dataset was split into 80% and 20% for training and testing respectively. Three modelling techniques, XGBoost, decision tree and logistic regression, were assessed for their relative performance in terms of precision, sensitivity and specificity.

**Results:**

There were 12471 unique patient encounters. Training was done on 10459 encounters and 39 data elements were included. When tested on 2012 encounters, the logistic regression model performed the best (86.7% sensitivity, 71.4% specificity). The model correctly classified 1377 out of 1388 (99.2%) encounters as “appropriate” (do not require ASP intervention). 624 antibiotic use encounters were classified as “inappropriate”, of which only 72 were truly inappropriate (positive predictive value for ASP intervention, PPV 11.5%). The low PPV was likely due to inadequate representation of “inappropriate” cases in the training dataset (4.1%). Applying this model would prioritise the number of immediate ASP reviews needed to identify cases for intervention by two-thirds, from 2012 to 624 (Figure 1).

Figure 1. Illustration of AI benefits in ASP

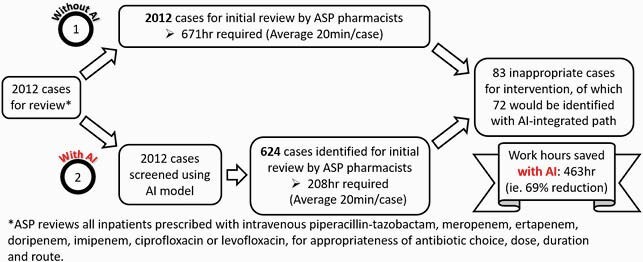

**Conclusion:**

ASPs can leverage on machine learning capabilities to improve audit efficiency. This can increase ASP’s productivity and staff’s job satisfaction as they are freed up to perform other work.

**Disclosures:**

**All Authors**: No reported disclosures

